# Prevalence of clinical characteristics of lipodystrophy in the US adult population in a healthcare claims database

**DOI:** 10.1186/s12902-024-01629-x

**Published:** 2024-07-02

**Authors:** Seonkyeong Yang, Caitlin Knox

**Affiliations:** 1 Department of Pharmaceutical Outcomes & Policy, College of Pharmacy, University of Florida, Gainesville, FL USA; 2 Regeneron Pharmaceuticals, Inc. Global Patient Safety, 777 Old Saw Mill River Road, Tarrytown, NY 10591 USA

**Keywords:** Lipodystrophy, Metabolic dysregulation, Adipose tissue, Metabolic complication, Clinical characteristic

## Abstract

**Background:**

Lipodystrophy (LD) is characterized by progressive loss of adipose tissue and consequential metabolic abnormalities. With new treatments emerging for LD, there is a growing need to understand the prevalence of specific comorbidities that may be commonly associated with the condition to contextualize its natural history without any disease-modifying therapy.

**Objective:**

To examine the risk of specific clinical characteristics in people living with LD in 2018–2019 compared with the general US population, among the commercially insured US population.

**Methods:**

A retrospective cohort study was conducted using the 2018–2019 Clinformatics® Data Mart database. An adult LD cohort (age ≥ 18 years) with ≥1 inpatient or ≥2 outpatient LD diagnoses was created. The LD cohort included non–HIV-associated LD (non–HIV-LD) and HIV-associated LD (HIV-LD) subgroups, which were compared against age- and sex-matched control groups with a 1:4 ratio from the general population with neither an LD or an HIV diagnosis using odds ratios (ORs) with 95% confidence intervals (CIs).

**Results:**

We identified 546 individuals with non–HIV-LD (mean age, 60.3 ± 14.9 years; female, 67.6%) and 334 individuals with HIV-LD (mean age, 59.2 ± 8.3 years; female, 15.0%) in 2018–2019. Compared with the general population, individuals with non–HIV-LD had higher risks (OR [95% CI]) for hyperlipidemia (3.32 [2.71–4.09]), hypertension (3.58 [2.89–4.44]), diabetes mellitus (4.72 [3.85–5.79]), kidney disease (2.78 [2.19–3.53]), liver fibrosis or cirrhosis (4.06 [1.66–9.95]), cancer (2.20 [1.59–3.01]), and serious infections resulting in hospitalization (3.00 [2.19–4.10]). Compared with individuals with HIV, those with HIV-LD have higher odds of hypertension (1.47 [1.13–1.92]), hyperlipidemia (2.46 [1.86–3.28]), and diabetes (1.37 [1.04–1.79]).

**Conclusions:**

LD imposes a substantial burden on affected individuals due to a high prevalence of metabolic comorbidities and other complications as compared with the general non-LD population. Future longitudinal follow-up studies investigating the causality between LD and observed comorbidities are warranted.

## Background

Lipodystrophy (LD) is a group of rare disorders of selective deficiency of adipose tissue affecting various areas of the body, and is associated with significant metabolic abnormalities [1]. LD is classified into generalized LD (GLD) and partial LD (PLD), which are characterized by near-complete loss or selective loss of adipose tissue, respectively. Both GLD and PLD are sub-divided into genetic (i.e. congenital GLD and familial PLD) and acquired subtypes (i.e. acquired GLD and acquired PLD) based on etiology [2, 3]. HIV-associated LD (HIV-LD) is a distinct subtype of acquired PLD, and is the most prevalent type of LD [4, 5]. Although abnormal fat distribution appears in varying presentations, HIV-LD tends to represent peripheral subcutaneous fat loss (i.e. lipoatrophy) with or without central fat accumulation (i.e. lipohypertrophy) [6, 7]. While the exact etiology of HIV-LD is still unknown, there is growing evidence indicating that HIV-associated lipoatrophy is associated with the older thymidine analog-containing highly active antiretroviral therapies stavudine and zidovudine [6, 8, 9]. The presentation of HIV-associated lipoatrophy is similar to that seen in non–HIV-associated LD (non–HIV-LD), while HIV-associated lipohypertrophy shares similar features with metabolic syndrome [10–12].

LD is commonly associated with metabolic comorbidities, including dyslipidemia, elevated triglyceride levels, insulin resistance/diabetes, metabolic dysfunction-associated steatotic liver disorder (MASLD), recurrent acute pancreatitis, autoimmune disorders, nephropathy, and reproductive dysfunction [13]. However, the literature has yet to explore the common comorbidities associated with LD as compared to the general population. Furthermore, with the emergence of new disease-modifying treatment options for LD, there is growing awareness of the need to understand the prevalence of specific clinical comorbidities commonly associated with LD, and to evaluate whether these are prevalent in the natural history of LD without disease-modifying treatment. Therefore, we aim to examine specific LD-associated clinical characteristics in a commercially insured US population using a nationwide administrative claims database between 2018 and 2019.

## Methods

### Data source and study design

We examined specific LD-associated clinical characteristics using the Clinformatics® Data Mart database from January 1, 2018, to December 31, 2019. Clinformatics Data Mart is an integrated US healthcare claims database that includes privately insured enrollees with commercial or Medicare Advantage plans affiliated with Optum. The database contains statistically de-identified and Health Insurance Portability and Accountability Act–compliant medical claims, including healthcare services performed in inpatient and outpatient settings, and pharmacy claims for approximately 15–20 million individuals annually. The Clinformatics Data Mart population is geographically representative, spanning all 50 US states. In addition to medical and pharmacy claims, the database includes provider data as well as information on patient enrollment and demographics [14]. Clinformatics Data Mart utilizes internal and external sources to comprehensively capture death information, such as the Death Master File maintained by the Social Security Office, enrollment data files in Medicare data, obituary data, facility claims, member coverage information, and Optum electronic health record data.

### Study population

**Fig. 1:**
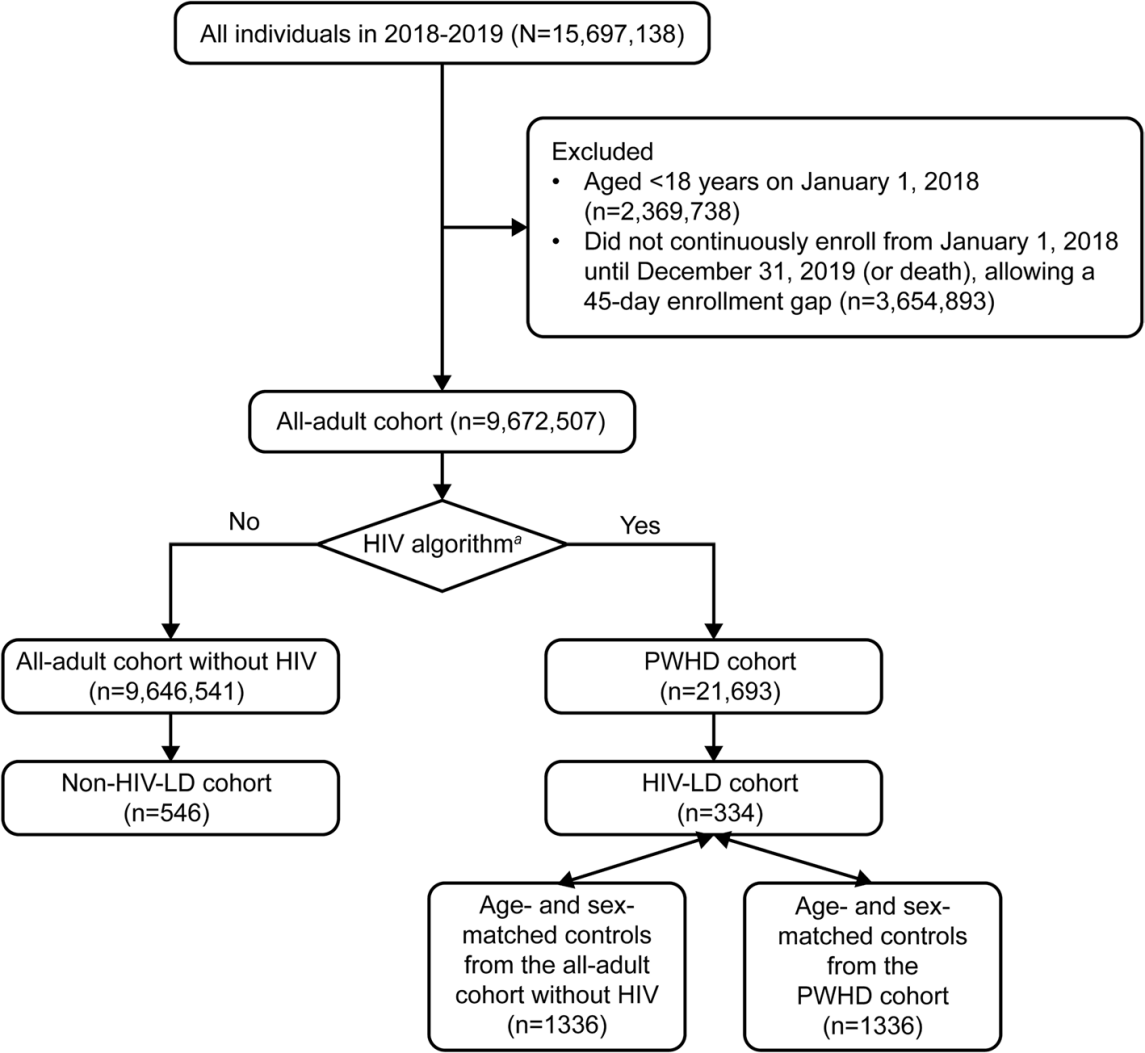
Flow chart for clinical characteristic analysis in 2018–2019. ^*a*^Individuals who had at least one inpatient or two outpatient HIV diagnoses (i.e. ICD-9-CM: 042, 079.53, V08; ICD-10-CM: B20, B97.35, Z21) on separate calendar dates are qualified as having HIV. Abbreviations: HIV-LD, lipodystrophy associated with HIV; ICD-9-CM, International Classification of Diseases, Ninth Edition, Clinical Modification; ICD-10-CM, International Classification of Diseases, Tenth Edition, Clinical Modification; non–HIV-LD, lipodystrophy not associated with HIV; PWHD, people living with HIV disease

First, we assembled an all-adult cohort, including all eligible adult individuals (aged ≥ 18 years on January 1, 2018) who continuously enrolled from January 1, 2018, until December 31, 2019 (or until they died, if this was before the end date of the study period), allowing a 45-day enrollment gap (Fig. 1). Next, a cohort comprising patients with a diagnosis of HIV disease was identified to include individuals who had at least one inpatient or two outpatient HIV diagnoses (i.e. International Classification of Diseases, Ninth Revision, Clinical Modification [ICD-9-CM]: 042, 079.53, V08; International Classification of Diseases, Tenth Revision, Clinical Modification [ICD-10-CM]: B20, B97.35, Z21) on separate calendar dates among the all-adult cohort. This identification algorithm was modified from the validated Medicaid-based algorithm to identify people living with HIV [15, 16]. An LD cohort was also created from the all-adult cohort by identifying LD diagnosis (i.e. ICD-9-CM: 272.6; ICD-10-CM: E88.1) in medical claims, or metreleptin prescription (National Drug Code: 66780-310-01, 76431-210-01) in pharmacy claims. In order to increase specificity and decrease misclassification due to rule-out diagnosis, at least one inpatient or two outpatient LD diagnoses on separate calendar dates were required. The LD cohort was further divided into non–HIV-LD and HIV-LD subgroups. We defined the non–HIV-LD group as individuals without any HIV diagnosis, and the HIV-LD group as individuals with at least one inpatient or two outpatient HIV diagnoses among the LD cohort. By definition, the non–HIV-LD group included all individuals with GLD or non–HIV-associated PLD, and the HIV-LD group included HIV-associated lipoatrophy and lipohypertrophy.

Individuals in the non–HIV-LD group were matched by age and sex in a 1:4 ratio to controls from the all-adult cohort without HIV. The controls were required to have neither an LD nor an HIV diagnosis. To minimize the effect of fat alterations associated with HIV infection and antiretroviral therapy, individuals in the HIV-LD group were matched by age and sex in a 1:4 ratio to controls from the cohort of people living with HIV. The people living with HIV controls were required not to have an LD diagnosis. Age and sex were selected as matching criteria due to their strong association with numerous chronic conditions. The 1:4 matching ratio was employed to enhance the statistical power of the analysis, supported by an adequate sample size for the control groups.

### Descriptive data analysis 2018–2019

We examined patient demographics (i.e. age, sex, and race/ethnicity), estimated their Elixhauser Comorbidity Index [17] (identification of 38 different pre-existing conditions based on secondary diagnoses), and estimated the prevalence of clinical characteristics among the non–HIV-LD and HIV-LD cohorts. The specific clinical characteristics evaluated included hyperlipidemia, diabetes mellitus, hypertension, acute myocardial infarction (AMI), MASLD or metabolic dysfunction-associated steatohepatitis (MASH), liver fibrosis or cirrhosis, acute pancreatitis, kidney disease (i.e. acute or chronic glomerulonephritis, acute or chronic renal failure, nephritis or nephrotic syndrome, renal failure, or proteinuria), autoimmune diseases (i.e. systematic lupus erythematosus [SLE], rheumatoid arthritis, or autoimmune thyroiditis), cancers excluding non-melanoma skin cancers, and serious infections (i.e. bacteremia, pneumonia, skin/soft tissue infection, gastrointestinal infection, acute osteomyelitis, acute pyelonephritis, or acute meningitis) resulting in hospitalization. We examined the prevalence of overall cancer and specific types of cancers (i.e. lymphoma, breast cancer, or prostate cancer). Lymphoma was identified with at least one inpatient diagnosis or two outpatient lymphoma diagnoses on separate calendar dates occurring at least 2 months apart. This approach was a modified version of a validated algorithm developed by Setoguchi et al., expecting > 80% sensitivity and > 90% specificity [18]. We also applied the same identification algorithm to other cancers [18]. To identify AMI, we applied an identification algorithm yielding 86.0% positive predictive value from a primary hospital discharge diagnosis of AMI [19]. Acute pancreatitis was identified from a primary hospital discharge diagnosis of acute pancreatitis [20]. To identify SLE, we modified a claims-based identification algorithm by using at least one inpatient diagnosis or two outpatient diagnoses at least 2 months apart [21].

Furthermore, we used a case definition of serious infections resulting in hospitalizations [22]. For hyperlipidemia, diabetes mellitus, and hypertension, we used the Centers for Medicare and Medicaid Services (CMS) Chronic Condition Warehouse (CCW) algorithms using at least one inpatient or two outpatient diagnoses on separate calendar dates [23].

### Statistical analysis

The patient demographics and clinical characteristics among non–HIV-LD and HIV-LD cohorts were presented as percentages for categorical variables, and as means and standard deviations for continuous variables. We compared demographics and clinical characteristics between the LD cohorts and matched controls using the Chi-square test, Fisher’s exact test, and student t-test, as appropriate. Logistic regression models were used to estimate the odds ratios (ORs) and accompanying 95% confidence intervals (CIs) for each clinical characteristic among non–HIV-LD and HIV-LD cohorts compared with the matched controls. All analyses were performed using Health Data software (Panalgo, Boston, MA, USA) with a two-sided level of statistical significance of 0.05.

### Data and resource availability

All data analyzed during this study are from the Clinformatics Data Mart database that is cited in the Methods section. All analyses conducted in this study are presented in the manuscript.

## Results

### Clinical characteristics of individuals with non-HIV-LD versus matched controls from the all-adult cohort without HIV

We identified 546 individuals with non–HIV-LD (mean age, 60.3 ± 14.9 years; female, 67.6%; non-Hispanic White, 53.8%) and 2184 age- and sex-matched controls from the all-adult cohort without HIV between 2018 and 2019. Individuals with non–HIV-LD were more likely to use healthcare services compared with matched controls as follows: having ≥ 2 hospitalizations (17.9% vs 4.3%; *P* < 0.001), having ≥ 2 emergency department visits (29.7% vs 12.0%; *P* < 0.001), and having ≥ 10 outpatient visits (93.6% vs 63.7%; *P* < 0.001) during the 2-year study period (Table 1). Compared to matched controls, individuals with non–HIV-LD had higher odds of having all of the identified metabolic comorbidities and complications (i.e. hypertension, hyperlipidemia, diabetes mellitus, MASLD/MASH, AMI, liver fibrosis or cirrhosis, and acute pancreatitis; Table 2). In addition, those with non–HIV-LD had increased odds of having kidney disease, identified autoimmune diseases, serious infections resulting in hospitalization, and any cancer, mostly breast cancer and prostate cancer (Table 2). The Elixhauser index was greater in individuals with non–HIV-LD (6.5 ± 4.1) than in matched controls (3.2 ± 3.5; *P* < 0.001; Table 1).

**Table 1:** Demographics and clinical characteristics of individuals with lipodystrophy and age- and sex-matched controls in 2018–2019

	**Non–HIV-LD individuals** ***n* = 546**	**Matched controls from the all-adult cohort without HIV** ***n* = 2184**	***P*-value**	**HIV-LD individuals** ***n* = 334**	**Matched controls from the people living with HIV cohort** ***n* = 1336**	***P*-value**
Demographics						
Age in years, mean (SD)	60.3 (14.9)	60.3 (14.9)	1.00	59.2 (8.3)	59.2 (8.3)	1.00
Age categories, years, %			1.00			1.00
18–34	5.7	5.7		0.6	0.6	
35–49	16.8	16.8		8.7	8.7	
50–64	31.1	31.1		63.5	63.5	
≥ 65	46.3	46.3		27.2	27.2	
Female, %	67.6	67.6	1.00	15.0	15.0	1.00
Race/ethnicity, %			< 0.001			0.003
Non-Hispanic Asian	4.6	4.1		1.8	1.5	
Non-Hispanic Black	20.0	9.0		17.4	26.0	
Non-Hispanic White	53.8	60.9		56.9	49.5	
Hispanic	9.2	11.0		14.1	10.6	
Unknown	12.5	15.0		9.9	12.4	
Insurance type, %			< 0.001			0.31
Commercial plan	31.9	47.9		43.4	40.2	
Medicare Advantage plan	68.1	52.1		56.6	59.8	
Healthcare utilization, %						
Number of hospitalizations			< 0.001			0.80
0	63.4	85.5		76.9	75.4	
1	18.7	10.2		13.8	15.2	
≥ 2	17.9	4.3		9.3	9.4	
Number of ED visits, %			< 0.001			0.51
0	46.2	72.1		60.8	57.3	
1	24.2	15.9		18.3	20.0	
≥ 2	29.7	12.0		21.0	22.8	
Number of outpatient visits, %			< 0.001			< 0.001
0–9	6.4	36.3		7.8	18.7	
≥ 10	93.6	63.7		92.2	81.3	
Clinical characteristics, %						
Metabolic comorbidities/complications						
Hypertension	73.8	44.0	< 0.001	68.6	59.7	0.004
Hyperlipidemia	69.4	40.6	< 0.001	75.7	55.9	< 0.001
Diabetes	53.7	19.7	< 0.001	31.1	24.9	0.02
MASLD/MASH	7.0	2.2	< 0.001	3.6	3.2	0.86
AMI	3.3	1.4	0.004	3.0	2.7	0.91
Liver fibrosis/cirrhosis	2.2	0.5	< 0.001	3.3	2.8	0.80
Acute pancreatitis	1.3	0.3	0.01	0.0	0.7	0.22
Autoimmune diseases						
Rheumatoid arthritis	4.9	1.8	< 0.001	0.9	0.9	1.00
Autoimmune thyroiditis	2.4	0.7	0.002	0.6	0.1	0.18
SLE	1.6	0.3	0.01	0.3	0.2	1.00
Cancer						
Any cancer^***a***^	12.8	6.3	< 0.001	10.2	8.7	0.45
Breast cancer	4.9	2.0	< 0.001	0.6	0.4	0.63
Prostate cancer	2.6	1.1	0.02	2.1	2.5	0.78
Lymphoma	0.5	0.5	1.00	1.5	0.9	0.36
Other						
Serious infections^***b***^ resulting in hospitalization	14.7	5.4	< 0.001	10.8	11.2	0.89
Kidney disease^***c***^	26.7	11.6	< 0.001	28.1	23.1	0.06
Elixhauser index	6.5 (4.1)	3.2 (3.5)	< 0.001	6.1 (3.7)	5.5 (3.8)	0.01

*Abbreviations: AMI* Acute myocardial infarction, *ED* Emergency department, *HIV-LD* Lipodystrophy associated with HIV, *LD* Lipodystrophy, *MASLD* Metabolic dysfunction-associated steatotic liver disease, *MASH* Metabolic dysfunction-associated steatohepatitis, *non–HIV-LD* Lipodystrophy not associated with HIV, *SD* Standard deviation, *SLE* Systemic lupus erythematosus

^a^Any cancer excludes non-melanoma skin cancer

^b^Serious infections include bacteremia, pneumonia, skin and soft tissue infection, gastrointestinal infection, acute osteomyelitis, acute pyelonephritis, and acute meningitis

^c^Kidney disease includes acute or chronic glomerulonephritis, acute or chronic renal failure, nephritis or nephrotic syndrome, renal failure, and proteinuria

**Table 2:** Odds ratios for the association between lipodystrophy and clinical characteristics for individuals with non-HIV-LD and HIV-LD compared to age- and sex-matched controls in 2018–2019

**Clinical characteristics, OR (95% CI)**	**Non–HIV-LD individuals vs matched controls from the all-adult cohort without HIV**^***a***^	**HIV-LD individuals vs matched controls from the** people living with HIV **cohort**^***b***^
Metabolic comorbidities/complications		
Hypertension	3.58 (2.89–4.44)	1.47 (1.13–1.92)
Hyperlipidemia	3.32 (2.71–4.09)	2.46 (1.86–3.28)
Diabetes	4.72 (3.85–5.79)	1.37 (1.04–1.79)
MASLD/MASH	3.40 (2.13–5.39)	1.12 (0.53–2.19)
AMI	2.45 (1.27–4.57)	1.11 (0.49–2.32)
Liver fibrosis/cirrhosis	4.06 (1.66–9.95)	1.16 (0.53–2.35)
Acute pancreatitis	4.04 (1.41–11.6)	—
Autoimmune diseases		
Rheumatoid arthritis	2.86 (1.67–4.84)	1.00 (0.18–3.73)
Autoimmune thyroiditis	3.30 (1.45–7.37)	4.01 (0.29–55.51)
Lupus erythematosus	5.21 (1.72–16.53)	1.33 (0.03–16.67)
Cancer		
Any cancer^*c*^	2.20 (1.59–3.01)	1.19 (0.77–1.80)
Breast cancer	2.59 (1.52–4.33)	1.60 (0.15–9.84)
Prostate cancer	2.27 (1.08–4.58)	0.82 (0.30–1.90)
Lymphoma	1.09 (0.19–4.15)	1.68 (0.46–5.15)
Other		
Serious infections resulting in hospitalization^*d*^	3.00 (2.19–4.10)	0.96 (0.63–1.42)
Kidney disease^*e*^	2.78 (2.19–3.53)	1.31 (0.99–1.72)

*Abbreviations: CI* Confidence interval, *HIV-LD* Lipodystrophy associated with HIV, *MI* Myocardial infarction, *MASLD* Metabolic dysfunction-associated steatotic liver disease, *MASH* Metabolic dysfunction-associated steatohepatitis, *non–HIV-LD* Lipodystrophy not associated with HIV, *OR* Odds ratio

^a^Individuals in the non–HIV-LD cohort were matched by age and sex in a 1:4 ratio to controls in the all-adult cohort without HIV. The controls were required to have neither an LD nor an HIV diagnosis

^b^Individuals in the HIV-LD cohort were matched by age and sex in a 1:4 ratio to controls in the people living with HIV cohort. The people living with HIV controls were required not to have an LD diagnosis

^c^Any cancer excludes non-melanoma skin cancer

^d^Serious infections include bacteremia, pneumonia, skin and soft tissue infection, gastrointestinal infection, acute osteomyelitis, acute pyelonephritis, and acute meningitis

^e^Kidney disease includes acute or chronic glomerulonephritis, acute or chronic renal failure, nephritis or nephrotic syndrome, renal failure, and proteinuria

### Clinical characteristics of individuals with HIV-LD versus matched controls from the people living with HIV cohort

We identified 334 individuals with HIV-LD (mean age, 59.2 ± 8.3 years; female, 15.0%; non-Hispanic White, 56.9%) and 1336 age- and sex-matched controls from the people living with HIV cohort between 2018 and 2019. Individuals with HIV-LD were more likely to have ≥ 10 outpatient visits compared to matched people living with HIV controls during the 2-year study period (92.2% vs 81.3%; *P* < 0.001); however, there were no statistically significant differences between those having ≥ 2 hospitalizations (9.3% vs 9.4%; *P* = 0.80) and those having ≥ 2 emergency department visits (21.0% vs 22.8%; *P* = 0.51; Table 1). Compared to matched people living with HIV, those with HIV-LD had higher odds of having hypertension (68.6% vs 59.7%; *P* = 0.004, OR: 1.47; 95% CI: 1.13–1.92), hyperlipidemia (75.7% vs 55.9%; *P* < 0.001, OR: 2.46, 95% CI: 1.86–3.28), and diabetes mellitus (31.1% vs 24.9%; *P* = 0.02, OR: 1.37, 95% CI: 1.04–1.79; Table 2). The Elixhauser index was greater in individuals with HIV-LD (6.1 ± 3.7) than in matched people living with HIV controls (5.5 ± 3.8; *P* = 0.01; Table 1). However, the prevalence of other clinical characteristics was comparable between those with HIV-LD and matched people living with HIV controls (Tables 1 and 2).

## Discussion

Our study yielded two important findings using a US nationwide cohort of the commercially insured adult population. First, we demonstrated the high prevalence of metabolic abnormalities and comorbidities in individuals with LD; and second, the findings may also shed light on the suspected association between immune dysregulation, cancer development, and LD.

The association between LD and metabolic comorbidities is well recognized in the literature. Our results were consistent with previous research, as we observed a high burden of metabolic comorbidities among individuals with non–HIV-LD, although due to the design of our study we were not able to explore the incidence of these comorbidities. Similar to the findings from the study by Gonzaga-Jauregui and colleagues [24], we observed higher ORs of having hypertension, hyperlipidemia, diabetes mellitus, and MASLD/MASH among individuals with non–HIV-LD compared with age- and sex-matched controls from the all-adult cohort without HIV. Our study also demonstrated the higher odds between non–HIV-LD and complications of metabolic comorbidities, such as AMI, liver fibrosis or cirrhosis, and acute pancreatitis, as compared to the general population. An increased risk of metabolic comorbidities can lead to accelerated coronary artery disease [25]. MASH is one of the leading causes of advanced liver disease, such as liver fibrosis and cirrhosis, and type 2 diabetes is identified as a strong risk factor for progressive liver disease [26–28]. In addition, hypertriglyceridemia is an infrequent but well-established cause of acute pancreatitis [29]. The aforementioned evidence indicates that individuals with LD are at higher risk of AMI, liver fibrosis or cirrhosis, and acute pancreatitis. Consistent with non–HIV-LD, individuals with HIV-LD are more likely to have hypertension, hyperlipidemia, or diabetes mellitus compared with age- and sex-matched controls from the people living with HIV cohort.

Some studies are suggestive of a potential association between LD and autoimmune diseases [30–32]. We observed higher odds of autoimmune diseases in the non–HIV-LD cohort, and higher odds were observed for rheumatoid arthritis, autoimmune thyroiditis, and lupus compared with the age- and sex-matched controls from the all-adult cohort without HIV.

We observed that individuals with non–HIV-LD and HIV-LD have an increased risk of cancer compared with matched controls. Specifically, individuals with non–HIV-LD are more likely to have breast cancer and prostate cancer, whereas individuals with HIV-LD had numerically higher odds of having lymphoma compared with the people living with HIV cohort; however, this was not statistically significant.

All studies utilizing secondary data sources have inherent limitations, and our study is no exception. We did not perform subgroup analyses for GLD and PLD, since there are no specific ICD-9/10-CM codes for LD subtypes, or validated algorithms currently available. However, we categorized LD into non–HIV-LD and HIV-LD using a combination of ICD-9/10-CM codes for LD and HIV, which is consistent with previous studies [24, 33]. Due to its rarity, heterogeneity, and similarity in disease manifestation with metabolic syndrome (particularly for PLD) [13], LD is commonly underrecognized and misdiagnosed in clinical practice. This may have contributed to the under-identification of LD cases in our study. However, the use of the ICD-9/10-CM codes provides a standard approach to identify the frequency of LD diagnosis in real-world settings. In addition, there is a potential for misclassification of comorbidities due to improper coding or rule-out diagnosis. Therefore, we conducted a thorough search to identify validated claims-based algorithms. Where no such algorithm existed, we adopted the “one inpatient or two outpatient diagnoses” approach to increase specificity and minimize misclassification resulting from rule-out diagnosis. This approach was also consistent with most CCW algorithms to identify chronic conditions in the CMS administrative claims data [23]. As a limitation inherent in cross-sectional studies, we did not investigate the temporal dynamic of clinical characteristics; thus, the findings of this study cannot be used to establish causality between LD and clinical characteristics. Additionally, we only adjusted for age and sex, which are significant risk factors for many chronic conditions, and we did not control for other sociodemographic factors or medications that have known associations with LD. However, it is still meaningful to understand the general prevalence of comorbidities in individuals with LD. Our primary objective was to measure the overall comorbidity burden rather than the causality between LD and specific chronic conditions, which requires strict confounder adjustment. To the best of our knowledge, this study is the first large claims-based study to explore clinical characteristics observed in patients with LD.

## Conclusions

In summary, although LD is a group of rare diseases, it imposes a substantially high burden on affected individuals, and has increased odds of a range of comorbidities and complications (metabolic abnormalities, autoimmune diseases, cancers, and serious infections resulting in hospital admissions). Future longitudinal follow-up studies are warranted to investigate the temporal dynamics between LD and observed clinical characteristics.

## Data Availability

All data analyzed during this study are from the Clinformatics^®^ Data Mart database that is cited in the Methods section. All analyses conducted in this study are presented in the manuscript.

## Abbreviations

AMI: Acute myocardial infarction
CMS: Centers for Medicare and Medicaid Services
CCW: Chronic condition warehouse
CI: Confidence interval
GLD: Generalized lipodystrophy
HIV-LD: HIV-associated lipodystrophy
ICD-9-CM: International Classification of Diseases, Ninth Edition Clinical Modification
ICD-10-CM: International Classification of Diseases, Tenth Edition, Clinical Modification
LD: Lipodystrophy
MASLD: Metabolic dysfunction-associated steatotic liver disease
MASH: Metabolic dysfunction-associated steatohepatitis
non–HIV-LD: non–HIV–associated lipodystrophy
OR: Odds ratio
PLD: Partial lipodystrophy
SLE: Systematic lupus erythematosus
